# Variables associated with subclinical atherosclerosis in a cohort of rheumatoid arthritis patients: Sex-specific associations and differential effects of disease activity and age

**DOI:** 10.1371/journal.pone.0193690

**Published:** 2018-03-01

**Authors:** Delia Taverner, Joan-Carles Vallvé, Raimón Ferré, Silvia Paredes, Lluís Masana, Antoni Castro

**Affiliations:** 1 Secció de Reumatologia, Servei de Medicina Interna, Hospital Universitari Sant Joan, Institut Investigació Sanitària Pere Virgili. Reus, Catalonia, Spain; 2 Facultat de Medicina, Universitat Rovira i Virgili, CIBER de Diabetes y Enfermedades Metabólicas Asociadas, Institut Investigació Sanitària Pere Virgili. Reus, Catalonia, Spain; 3 Facultat de Medicina, Universitat Rovira i Virgili, Servei de Medicina Interna, Hospital Universitari Sant Joan, CIBER de Diabetes y Enfermedades Metabólicas Asociadas, Institut Investigació Sanitària Pere Virgili. Reus, Catalonia, Spain; Keio University, JAPAN

## Abstract

**Objective:**

To advance the study of variables associated with subclinical atherosclerosis in rheumatoid arthritis (RA) with special consideration for the degree of disease activity, age and gender.

**Methods:**

The carotid intima-media thickness (cIMT) and the presence of carotid atherosclerotic plaques along with clinical and biochemical characteristics were determined in 214 RA patients.

**Results:**

Adjusted analysis reveals that men had a 0.059 mm significantly increased cIMT compared with women (p = 0.001; R^2^ = 3.8%) and that age was associated with cIMT (β = 0.0048 mm; p = 0.0001; R^2^ = 16%). Interestingly, we observed a significant interaction between gender and age. Thus, the effect of age on cIMT was significantly increased (12%) in men compared with women (p-value for interaction term = 0.041). Moreover, adjusted multivariable linear regression analysis revealed that disease activity score (DAS28) was significantly associated with cIMT in women (β = 0.021; p = 0.018: R^2^ = 0.03) but not men. In particular, women with high disease activity had a 0.079 mm increased cIMT compared with women in remission (p = 0.026). In addition, men in remission had a 0.134 mm increased cIMT compared with women in remission (p = 0.003; R^2^ = 8.7%). Active patients did not exhibit differences in cIMT values. Furthermore, 43% of patients presented carotid plaques. The variables independently associated with carotid plaques were age, smoking, health assessment questionnaire, erythrocyte sedimentation rate and rheumatoid factor (p<0.0001; R^2^ = 46%).

**Conclusion:**

In our cohort of patients with RA, DAS28 and age are differentially associated with cIMT in men and women. Our findings could explain the contradictory results that have previously been published in the literature.

## Introduction

Rheumatoid arthritis (RA) is a systemic autoimmune disease that mainly affects synovial joints and causes chronic pain, bone erosion and progressive disability. With a prevalence of up to 0.5–1% of the general population, RA is the most common chronic inflammatory disease [1,2]. In addition to joint involvement, a high prevalence of comorbidities and an increase in cardiovascular (CV) risk characterizes RA patients compared with the general population [1,3]. Epidemiological studies describe a relative risk of developing a CV episode of 1.5 compared with non-RA controls [2]. RA can be considered an independent risk factor for developing CV disease (CVD), with its prevalence being as high as in patients with type 2 diabetes mellitus [4] and leading to an increase in the standardized mortality rate of up to 3.82 for acute myocardial infarction in the United States [5]. The chronic inflammatory state of RA seems to contribute to the development of accelerated atherosclerosis because the inflammatory process in the synovial membrane and atherosclerotic plaques share similar characteristics [6].

The assessment of RA patients with subclinical atherosclerosis by means of ultrasonographic determination of the carotid intima-media thickness (cIMT) and the presence of carotid plaques has been accepted as a good predictor of CV events [7]. However, the relationship between subclinical arteriosclerosis and RA is complex. Increased cIMT reflects a pre-atherogenic condition [8,9]; however, numerous CV risk factors that have causal relationships with atherosclerosis do not seem to fully explain the accelerated atherosclerosis these patients exhibit. Different studies have evaluated the relationship between subclinical arteriosclerosis (cIMT and presence of carotid plaques) and RA. Although recent studies have argued for the increase in cIMT and the prevalence of plaques in RA patients compared with controls [10–15], other articles have been published in which this association is not demonstrated [16–19]. The discrepancy between these results is likely explained by the low activity of the included patients because the degree of disease activity is associated with cIMT [11], which has been postulated to be a limiting factor in the study of the relationship between subclinical arteriosclerosis and RA. In addition, age, which is clearly associated with an increase in cIMT in different populations [20,21], has a more complex relationship in RA. For example, the group of patients with early RA, in which younger patients prevail, presents the greatest difference in cIMT compared with that of controls [10]. Thus, in RA, complex associations are noted between the degree of disease activity, age and cIMT. In addition, to date, no records are available in the literature evaluating the relationships between these associations and the gender of RA patients. Thus, the objective of the present study was to advance the study of the associations between subclinical arteriosclerosis and RA by specifically considering the relationship among age, gender and degree of disease activity of RA patients.

## Materials and methods

### Patients

The study population included all consecutive patients who visited via external consultations of the University Hospital Sant Joan de Reus and who fulfilled the diagnostic criteria of RA proposed by the American College of Rheumatology of 1987. We excluded patients older than 80 years and younger than 20 years and those with acute intercurrent diseases. Consistent with our Institution’s guidelines and the Helsinki Declaration, subjects were informed of the research nature of the study and provided written consent prior to participation. The study was approved by the clinical research Ethics Committee of the University Hospital Sant Joan. Patients were visited between the period of September 2011 and November 2014, and we performed blood collection and carotid ultrasound on the same day of the medical visit.

### Laboratory measurements

Blood samples were collected from 214 patients who had fasted for at least 12 hours. Analytical determinations included the following: haemogram, general biochemistry, haemoglobin glycoside, thyrotropin, albumin, lipid profile [triglycerides (TG), total cholesterol (TC) and low-density lipoprotein cholesterol (LDLc), high-density lipoprotein cholesterol (HDLc) and very-low-density cholesterol (VLDLc)] by enzymatic methods; apolipoproteins A1 and B by immunonephelometry; lipoprotein (a) by an enzyme-linked immunosorbent assay; and rheumatoid factor (RF), citrullinated anti-cyclic peptide antibodies (ACPA), antinuclear antibodies and inflammatory markers [erythrocyte sedimentation rate (ESR), C-reactive protein (CRP) and fibrinogen] by conventional methods. RF positive (RF+) was defined for values of RF>20, and positive ACPA (ACPA+) was defined for ACPA values>1.

### Clinical evaluation

The presence of CV risk factors (smoking, hypertension, diabetes and hypercholesterolemia) and histories of previous CV events and the use of lipid lowering drugs, antidiabetic agents or antiplatelet drugs were collected. In addition to joint physical examinations of RA, measurements of body weight, height, body mass index (BMI), waist circumference (WC), systolic blood pressure (SBP) and diastolic blood pressure (DBP) were obtained.

As a measure of disease activity, the disease activity score (DAS28) was calculated according to the ESR. Pain was measured using the 0–10 visual analogue scale, and any disability was reported by the patient using the health assessment questionnaire (HAQ) index. The DAS28 variable was categorized as remission (DAS28<2.6), low activity (2.6≤DAS28<3.2), moderate activity (3.2≤DAS28≤5) and high activity (DAS28>5).

### Carotid intima-media thickness

To measure cIMT, we used a My Lab 50 X-Vision sonograph (Esaote SpA, Barcelona, Spain) with a linear array ultrasound probe small parts broadband transducer (5–12 MHz). We identified and digitally recorded the far wall of the common carotid artery (1 cm proximal to the bifurcation) and the internal carotid artery (1 cm distal to the bifurcation) of the left and right carotid arteries. cIMT measurements were performed at the predefined points using ThickSoft image processing software (%). To reduce observer variability, a single operator obtained and measured the images. We averaged the measurements of three static images of left and right carotid arteries to obtain the mean cIMT. Carotid artery plaque was defined according to Mannheim consensus [22] as a focal structure that encroaches into the arterial lumen of at least 0.5 mm or 50% of the surrounding cIMT value or demonstrates a thickness >1.5 mm as measured from the media-adventitia interface to the intima-lumen interface.

### Statistical analysis

All data were analysed using the statistical software SPSS, version 23. Continuous variables are presented as the mean (standard deviation) and categorical as the percentage (number of individuals). ANOVA was used to evaluate differences between groups followed by Bonferroni correction as a post hoc test. For categorical variables, the differences between the proportions were analysed using the chi-Squared test. Bivariate correlations were estimated using the Pearson correlation coefficient r. To estimate the cIMT variable, multiple linear regression was used with multivariate models. Multivariate logistic regression was used to estimate the presence of carotid plaques. We initially selected clinical relevant variables and known confounders for inclusion in the multivariable logistic regression analysis. The iterative process of variable selection was performed using stepwise logistic regression analysis. A p-value<0.05 was considered statistically significant.

## Results

A total of 214 RA patients were included in the study. Table 1 presents the general characteristics of patients overall and based on gender. Men had significantly increased WC, SBP and DBP and reduced HDLc levels compared with females. Sixty percent of the patients were hypertensive, which was more frequently observed in men (73%) compared with women (52%), and women had significantly increased HAQ, ESR, and DAS28 index values than males (Table 1). Furthermore, the percentages of women in remission or with low, moderate or high disease activity were different from those for men (Table 1). A total of 75% of the patients received disease-modifying antirheumatic drugs, which included 20%, 57% and 51% of patients receiving biological drugs, non-steroidal anti-inflammatory drugs and/or corticosteroids, respectively, with no differences between sexes.

**Table 1:** General characteristics of RA patients overall and stratified by gender.

			RA (n = 214)	Female (n = 138)	Male (n = 76)	P value
**Characteristics of the groups**						
	Gender-female (%, n)		64.5 (138)			
	Age (years)		58(12)	57 (12)	59 (12)	0.55
	Body mass index (kg/m^2^)		27.8 (5.9)	27.7 (6.6)	28.1 (4.4)	0.62
	Waist circumference (cm)		93 (15)	88 (15)	100 (12)	<0.001
	Systolic blood pressure (mmHg)		137 (21)	135 (21)	142 (21)	0.024
	Diastolic blood pressure (mmHg)		81 (12)	80 (13)	84 (12)	0.025
	LDL cholesterol (mg/dL)		119 (31)	118 (31)	120 (31)	0.75
	HDL cholesterol (mg/dL)		66 (19)	72 (18)	54 (15)	<0.001
	Triglycerides (mg/dL)		105 (55)	102 (54)	112 (58)	0.21
	Glucose (mg/dL)		95 (23)	94 (25)	96 (18)	0.35
	Current smoker (%, n)		26.2(56)	27.5(38)	23.7(18)	0.34
	Hypertension (%, n)		60.3 (129)	53 (73)	74 (56)	0.003
	Diabetes mellitus (%, n)		11.7 (25)	10.9 (15)	13.2 (10)	0.62
	Dyslipidaemia (%, n)		41.1(88)	39.1 (54)	44.7 (34)	0.425
**Disease features**						
	Disease duration (years)		9.4 (9.1)	10.1 (9.9)	8.2 (7.5)	0.12
	DAS28		3.5 (1.3)	3.7 (1.3)	2.98 (1.1)	<0.001
		Disease remission (%, n)	27.1 (58)	20.3 (28)	39.5 (30)	<0.001
		Low disease activity (%, n)	18.7 (40)	14.5 (20)	26.3 (20)	
		Moderate disease activity (%, n)	44.4 (95)	52.2 (72)	30.3 (23)	
		High disease activity (%, n)	9.8 (21)	13 (18)	3.9 (3)	
	HAQ		0.45 (0.52)	0.59 (0.56)	0.21 (0.34)	<0.001
	Rheumatoid factor+ (%, n)		72.4 (155)	71.7 (99)	73.7 (56)	0.76
	ACPA+ (%, n)		81.3 (174)	83.3 (115)	77.6 (59)	0.31
	ESR (mm/h)		37 (26)	40 (27)	32 (22)	0.019
	CRP (mg/dL)		0.72 (0.83)	0.73 (0.80)	0.70 (0.88)	0.83
	Fibrinogen (mg/dL)		443 (97)	442 (96)	444 (100)	0.91
**Treatments (%, n)**						
	DMARDs		75.2 (161)	71.7 (99)	81.6 (62)	0.11
	Biological agent		20.1 (43)	23.2 (32)	14.5 (11)	0.13
	NSAIDs		57 (122)	57.2 (79)	56.6 (43)	0.92
	Corticosteroids		50.9 (109)	53 (73)	47 (36)	0.44

HAQ = health assessment questionnaire index, ACPA = citrullinated anti-cyclic peptide antibodies, ESR = erythrocyte sedimentation rate, CRP = C-reactive protein, DAS28 = disease activity score, DMARDs = disease-modifying antirheumatic drugs, NSAIDs = non-steroidal anti-inflammatory drugs.

**Fig 1:**
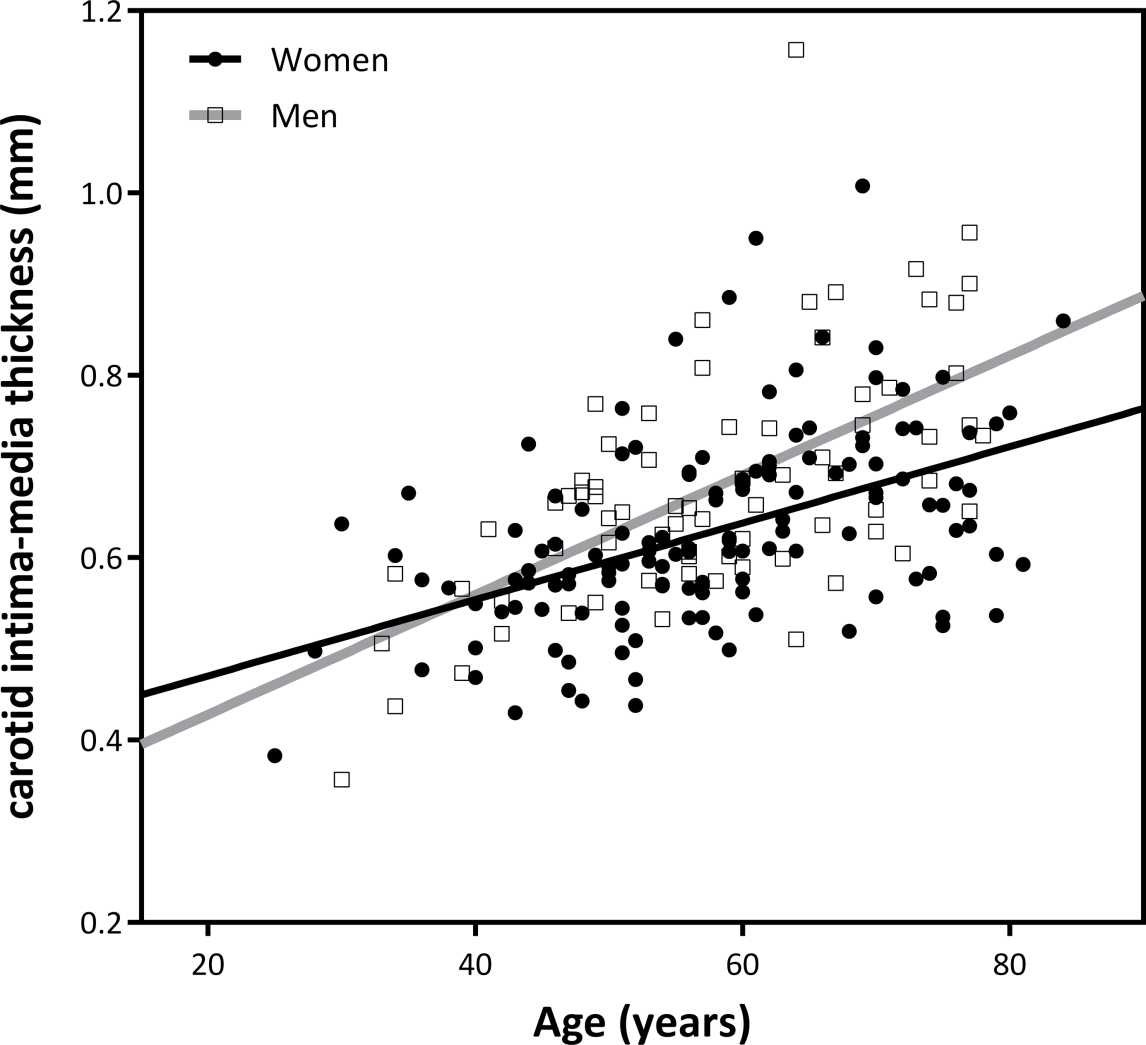
Correlation between age and carotid intima-media thickness (cIMT) by gender.

Crude analysis of the ultrasonographic variables showed, as expected, that age was highly correlated with cIMT (r = 0.53; p = 0.0001) and that men presented significantly larger values of cIMT than women (0.678 mm vs 0.627 mm; p = 0.005) (Table 2). After adjusting for traditional confounders and treatments (see Table 2), we quantified these associations and showed that men had a 0.059 mm larger cIMT than women (p = 0.001) and that the gender variable explained a significant 3.8% of the cIMT variability. Moreover, we showed a significant 0.0048 mm increase in cIMT for every year of increase in age (Table 2) and that the age variable explained a significant 16% of the cIMT variability (Table 2). Interestingly, we observed a significant interaction between age and gender (p value for interaction term = 0.041); the correlation between age and cIMT was 12% significantly steeper in men than in women (Fig 1). In particular, cIMT increased 0.0065 and 0.0043 mm for each year increase of age in men and women, respectively. Because of this interaction, further analyses were stratified by gender.

**Table 2:** Association of gender and age with sonographic measurements.

**A. Sonographic measures**				**RA**	**Female**	**Male**	**p-value**
	1-cIMT (mm)			0.645 (0.117)	0.627 (0.15)	0.678 (0.129)	0.005
	2-Plaque presence (%, n)			43.5 (93)	36.2 (50)	56.6 (43)	0.004
**B. cIMT**			**Coefficients**			**R**^**2**^ **(%)**	**p-value**
			**β**	**SE**	**95% CI**		
	Adjusted model					37	0.0001
		Gender	0.059	0.017	0.025–0.093	3.8	0.001
		Age	0.0048	0.00067	0.0035–0.0061	16	0.0001

(A1). Mean cIMT values in overall RA patients and divided by gender. ANOVA statistics for gender association with cIMT. (A2). Percentage of plaque presence in overall RA population and divided by gender. Chi-squared statistics for gender association with plaque presence. (B). Adjusted β linear regression estimates of the effect of age and male gender on cIMT applied to the overall population. The model is adjusted for disease duration, body mass index, ischaemic heart disease, ictus, peripheral artery disease, creatinine, hypertension, dyslipidaemia, type 2 diabetes mellitus, disease-modifying antirheumatic drugs, biological agents, corticosteroids, and non-steroidal anti-inflammatory drugs.

SE = standard error.

**Fig 2:**
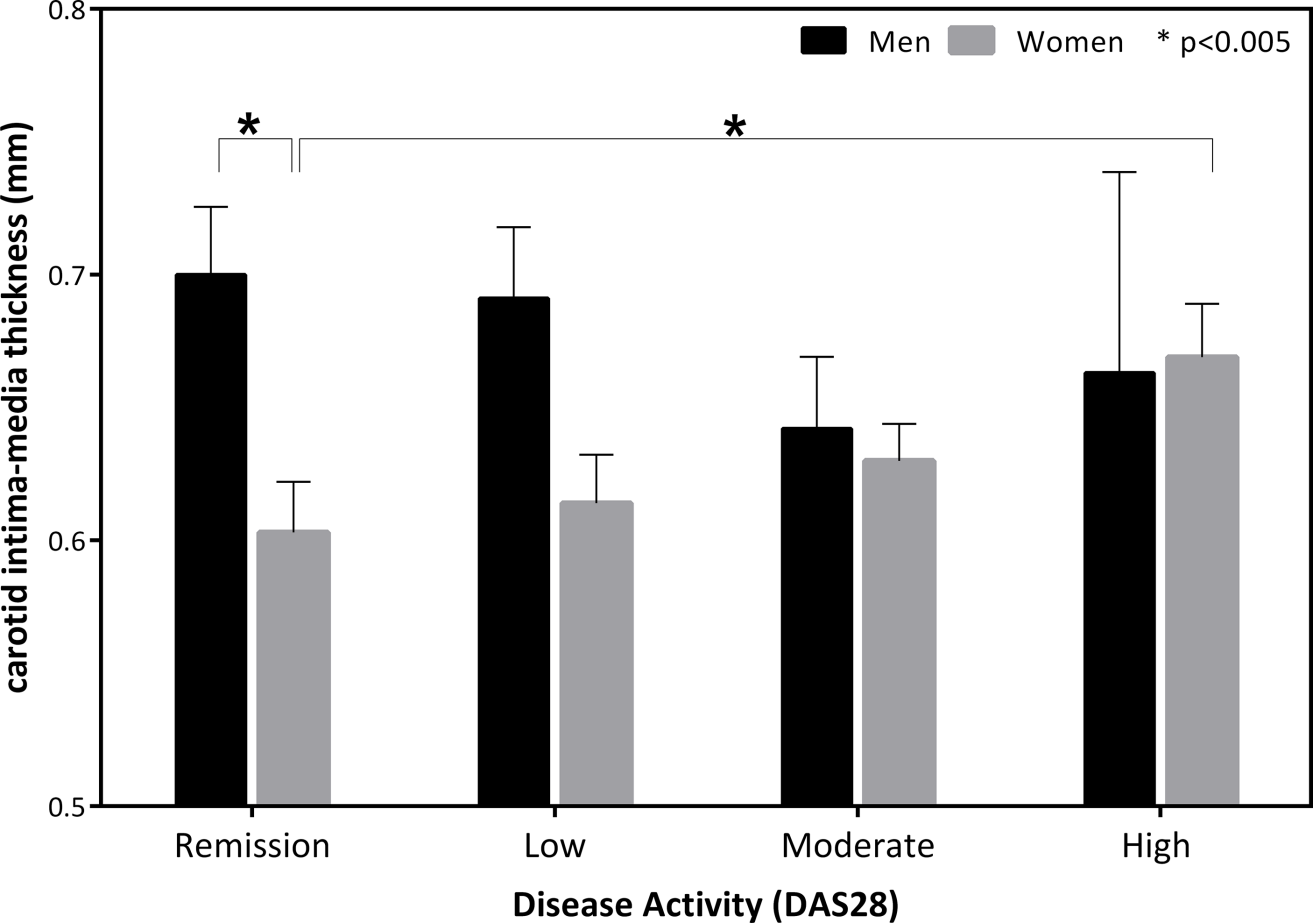
Effects of degrees of disease activity on carotid intima-media thickness (cIMT) by gender.

Adjusted multivariable linear regression analyses revealed that DAS28 was significantly associated with cIMT in women (β = 0.021; p = 0.018) (Table 3) and that DAS28 significantly explained 3% of cIMT variability. No association was observed between DAS28 and cIMT in men. When studying the effect on cIMT of DAS28 categorized in degrees of disease activity, adjusted analyses revealed that women with high disease activity had a 0.079 mm larger cIMT than women in remission (p = 0.026). No differences were observed either among the other categories or in men (Fig 2). In addition, the difference in cIMT observed between men and women was due to patients in remission because significant differences were observed in cIMT values in this group, with men in remission having a 0.134 mm higher cIMT compared with women in remission (p = 0.003). In this group, gender explained a significant 8.7% of the cIMT variability. Active patients did not exhibit differences in cIMT values (Fig 2). No other clinical or analytical variable related to RA disease exhibited a significant association with cIMT.

**Table 3:** Association of DAS28 with cIMT.

DAS28	Men	Women
**β coefficient**	0.0043	0.021
**SE**	0.015	0.0085
**95% CI**	-0.026–0.035	0.0035–0.037
**R**^**2**^ **(%)**	0.1	3
**p-value**	0.78	0.018

Adjusted β linear regression estimates of the effect of DAS28 on cIMT applied to men and women. The model is adjusted for disease duration, body mass index, ischaemic heart disease, ictus, peripheral artery disease, creatinine, hypertension, dyslipidaemia, type 2 diabetes mellitus, disease-modifying antirheumatic drugs, biological agents, corticosteroids, non-steroidal anti-inflammatory drugs and age. SE = standard error.

Next, regarding the presence of carotid artery plaques in RA patients, we observed that 43% had carotid plaques, with a significantly increased prevalence in men compared with women (Table 2). Furthermore, stepwise logistic regression analysis revealed that the variables significantly associated with the presence of atherosclerotic plaques were age, smoking, HAQ, RF+, and ESR. Table 4 presents the odds ratio (OR) associated with each of these variables. Age, smoking, ESR, and RF+ were associated with an increased likelihood for the presence of plaques, whereas HAQ was associated with a reduction in this probability. All these variables explained 40% of the plaque presence variability.

**Table 4:** Estimates of the effect of significant variables on plaque presence.

Significant variables	OR	95% CI		R^2^ (%)
Age	1.094	1.05	1.14	40
Smoking	3.66	1.68	8.03	
RF+	2.29	1.02	5.14	
HAQ	0.34	0.15	0.75	
ESR	1.017	1.00	1.03	

Adjusted OR estimates were assessed using stepwise logistic regression analysis applied to the overall population. R^2^ is calculated for all five variables.

HAQ = health assessment questionnaire index, ESR = erythrocyte sedimentation rate, and RF = rheumatoid factor.

## Discussion

In our study, we evaluated the relationship of cIMT with disease activity in conjunction with age and gender in a sample of 214 patients with RA. We observed a complex relationship among these variables and cIMT. In particular, we observed that males with RA had increased cIMT values compared with women, but these differences were due to patients in remission. As the disease activity increased, these differences disappeared, and the cIMT values of women became similar to those of men. In addition, women with high disease activity exhibited significantly increased cIMT values compared with women in remission; this result was not observed in men. Although different studies have evaluated the association between DAS28 and cIMT, the results are inconclusive. Thus, both studies with significant associations [7,23] and those with no associations between these variables have been described [24]. However, in contrast to our study, these studies did not evaluate the effect of gender among these associations, which could explain the discrepancies between studies. In our study, DAS28 was significantly associated with cIMT in women, significantly explaining 3% of the cIMT variability. Our results and others have demonstrated significantly higher DAS28 and HAQ values in females than in males, which could be justified both by the greater perception of pain and reduced muscle strength of females and the overestimation of functional capacity in males [25]. Furthermore, low DAS28 has been suggested as the cause of the lack of differences in cIMT between RA patients and controls that is observed in some studies [16,18,19].

In addition, we observed a significant interaction between age and gender. We observed a 12% significantly more pronounced correlation of age with cIMT in men compared with women. To the best of our knowledge, these data are reported for the first time. These results suggest a more rapid rate of cIMT thickening in men with RA than in women as age increases. The adjusted model (Table 2B) for our entire population of RA patients significantly explains a maximum of 37% of the variability of cIMT; thus, there is room for progress in the evaluation of new markers associated with cIMT in these patients.

In our study, we observed no significant association between the inflammatory variables (CRP, ESR and fibrinogen) and serological markers (RF+, ACPA+) studied and cIMT. Several publications have reported significant associations between these variables [8,10,26,27,23,28–34]. For instance, Ruscitti et al. reported that inflammatory burden together with systemic arterial hypertension, metabolic syndrome, and lack of clinical response are significantly correlated with an increased risk of developing new subclinical atherosclerosis [35,36]. However, negative results have also been reported, and low levels of inflammation have been suggested to be due to a timely assessment of inflammation that would not adequately reflect the fluctuating levels of inflammation characteristic of RA and the interactions of drugs as potential causes of these results [4,13,37–41].

The association between classical lipid profile values (LDLc, TG, and HDLc) and subclinical atherosclerosis is not well determined. Although positive associations have been reported [42]; other studies, including our own, have not reported such associations [16,43]. Given the evidence that inflammation is associated with both quantitative and qualitative changes in lipoproteins [44–46], static measures of classical lipid values might not be sufficient, and advanced measures (i.e., sizes and concentrations of lipid subfractions, compositional changes and functionality) are necessary to provide clinically useful information.

The presence of carotid plaques is considered a good predictor of CV risk. In our study, 43% of RA patients had carotid plaques, which is slightly higher than the 33% described in a meta-analysis of 35 studies [10]. In addition, variables significantly associated with the presence of atherosclerotic plaques in our RA patients were age, smoking, HAQ, ESR and RF+. These results do not disagree with different published studies [8,47,48]; however, discordant results are also presented [24,49]. Surprisingly, unlike previously described values [8,50], the HAQ values in our RA patients correlated positively with a reduced risk of presenting atherosclerotic plaques. This result could be explained by the cross-sectional design of the study, the lack of radiographic analysis of the damage or the subjective nature of this parameter.

The observed differences between some of the variables that are associated with cIMT and the presence of plaques could be explained if we take into account that cIMT and atherosclerotic plaques reflect different stages and pathophysiological processes of atherosclerosis [51,52]. Thus, cIMT represents the thickening of the muscular layer of the media, whereas plaque formation is a result of the thickening of the intima. In addition, plaque formation is considered a late stage in the atherosclerotic process, progressing from the initial fatty streak to an advanced atheromatous plaque [53].

cIMT is an independent predictor of cardiovascular events and for this reason it has been considered as a tool to improve the estimation of cardiovascular risk. However, its application in the clinical practice is still a matter of debate and is limited by the lack of positive results in interventional studies and clinical trials aimed to correlate cIMT regression with the improvement in cardiovascular survival.

In conclusion, in our cohort of patients with RA, disease activity measured according to DAS28 and age are associated with cIMT differentially in men and women. Our results may help to explain why DAS28 is not always associated with subclinical atherosclerosis. In addition, gender differences influencing cIMT could be justified by the increased incidence of RA in women and by factors intrinsic to the female sex that are external to the autoimmune disease itself (i.e., genetic and hormonal factors) and could help clinicians to manage these patients.

## Supporting information

S1 File Data set. (TXT)
